# Estimation of the Knee Joint with Single-Camera Smartphone

**DOI:** 10.3390/s26072148

**Published:** 2026-03-31

**Authors:** Michela Russo, Carlo Ricciardi, Maria Romano, Vittorio Santoriello, Alfonso Maria Ponsiglione, Francesco Amato, Maria Francesca Spadea

**Affiliations:** 1 Department of Chemical, Material and Industrial Production Engineering, University of Naples Federico II, 80125 Naples, Italy; 2 Department of Electrical Engineering and Information Technology, University of Naples Federico II, 80125 Naples, Italy; 3 Institute of Biomedical and Neural Engineering, Reykjavik University, 102 Reykjavik, Iceland; 4 Institute of Biomedical Engineering, Karlsruhe Institute of Technology, 76131 Baden-Württemberg, Germany

**Keywords:** gait analysis, video-based analysis, markerless motion capture, wearable sensors, smartphone single-camera, signal processing

## Abstract

(1) Background: Gait analysis provides quantitative information on walking patterns and has proven invaluable for assessing motor function in rehabilitation programmes. A markerless motion capture system combining computer vision techniques provides low-cost, real-time, portable gait analysis. (2) Methods: The kinematics of the knee and ankle of twenty-seven healthy volunteers were assessed using a single smartphone camera combined with the MediaPipe human pose estimation framework. The system was validated using the OPAL wearable sensor system by APDM Wearable Technologies. (3) Results: Findings showed close correspondence between the two systems for knee kinematics showing a mean absolute error of 4.10° ± 2.32° and 3.15° ± 3.10° for right and left knee flexion, respectively, and a mean absolute error of 2.30° ± 2.01° and 3.12° ± 2.63° for right and left knee extension, respectively. The mean absolute error for right and left knee range of motion was found to be 4.55° ± 3.12° and 4.15° ± 3.01°, respectively. Moreover, Bland–Altman plots indicated minimal bias (average 0.6 for flexion, average 0.47 for the extension, and 0.30 for the range of motion) and excellent correlation for knee flexion bilaterally (0.916 and 0.845 for the right and left side, respectively), with slightly lower but still satisfactory agreement for knee extension (0.862 and 0.845 for the right and left side, respectively). Conversely, ankle measurements revealed poor concordance: dorsiflexion and range of motion presented significant differences and systematic errors, while plantarflexion showed no statistical difference but weak correlation. (4) Conclusions: This study demonstrated that combining a smartphone camera with a human pose estimation framework allows for low-cost, real-time, portable gait analysis, particularly of the knee joint.

## 1. Introduction

Gait analysis is a non-invasive and systematic assessment of biomechanical locomotion. It provides quantitative elements about gait patterns, with the aim of understanding locomotion abnormalities and supporting clinicians in monitoring rehabilitation and making clinical decisions regarding neuromotor disorders or gait impairments [1,2,3]. Gait analysis focuses on three main categories of information: spatio-temporal parameters, which describe the rhythm walking; kinematics, which describe joint displacements in the anatomical planes; and kinetics, which describe the forces and moments underlying the dynamics of walking [4,5]. Originally developed as an academic discipline, gait analysis is increasingly being used in clinical practice as a tool for several purposes, such as in surgery, bracing, prosthetics and rehabilitation programmes, and for comparing the outcomes of different treatments [3,6,7]. When combined with specific rehabilitation programs, gait analysis can be used to evaluate the impact of interventions on motor function.

This approach is widely used to manage musculoskeletal disease, for example in case of knee osteoarthritis or post-surgery programmes following interventions such as knee arthroplasty [8]. Changes in mechanical loading at the knee joint can contribute to musculoskeletal pain, promote the onset of joint disease and worsen with age.

For this reason, assessing knee joint biomechanics is often used to inform clinical decisions in treatment planning [9]. Abid et al. [10], in a recent 2021 review, conducted an in-depth study of the biomechanics of the knee joint to classify knee disorders. The aim was to identify a biomechanical gait pattern that could be used as a diagnostic tool to assess conditions such as knee joint injuries and disorders. Knee joint injuries and pathologies that can affect gait include ligament and muscle injuries, femoral syndrome and osteoarthritis. Several osteoarthritis studies have recognised the importance of studying this aspect for two reasons: knee joints are the most commonly injured joints and common diagnostic methods, such as clinical evaluations, X-rays and MRIs, do not provide objective information on the knee joint’s functionality [11]. Biomechanical studies therefore provide quantitative information to supplement common physical assessments and enable more accurate diagnoses, particularly through the analysis of knee joint kinematics in the sagittal plane, which is fundamental. Several studies have demonstrated the excellent repeatability of kinematic data in the sagittal plane in maintaining postural stability during walking [12].

Beyond musculoskeletal applications, gait analysis has also proven valuable in neurological rehabilitation, where it supports interventions aimed at addressing motor impairments, including physical therapy, exercise and gait training [13,14]. In neurological disorders such as Parkinson’s disease (PD), gait analysis is increasingly employed to identify motor biomarkers associated with specific clinical features, including mild cognitive impairment [15,16,17,18], showing a reduced speed, stride length, cadence and range of motion (ROM) for joint movement in the sagittal plan, or provides objective measures to monitor disease progression and to evaluate treatment efficacy [19,20,21].

The optoelectronic system is considered as the gold standard for human gait analysis [22]. It captures human movement in the indoor environment using markers placed in specific areas of the body of the subject [23]. Advances in gait assessment technology have led to the development of wearable sensors, particularly Inertial Measurement Units (IMUs), which have emerged as a valuable and effective alternative to optoelectronic systems for measuring spatio-temporal and kinematic parameters [24,25]. In particular, IMUs, equipped with accelerometers, gyroscopes, and magnetometers, allow gait analysis to be performed outside of a traditional and calibrate gait laboratory [26]. IMUs require only the placement of wearable sensors, such as on the upper and lower limbs, which allows estimation of the subject’s position in space as well as the evaluation of 3D angular joint movements.

Recent studies have shown that IMUs can be used to evaluate gait patterns, particularly in the context of investigating joint kinematics [27,28,29]. Furthermore, IMUs can be used in home settings, where patients may feel more comfortable than in traditional clinical environments [30,31]. Ricciardi et al. [27] showed that the OPAL System (an IMUs system supplied by APDM Wearable Technologies, Inc., Portland, OR, USA) compared with an optoelectronic system for evaluating gait spatio-temporal parameters in patients with progressive supranuclear palsy revealed a perfect agreement for speed and a very close agreement for cadence and cycle duration. In the same way, Chia et al. [28] validated IMUs against optoelectronic motion capture systems, demonstrating a good to excellent correlation and a bias of less than 2° when estimating knee sagittal-plane ROM. In the similar manner, Zhang et al. [29] evaluated a custom IMU in patients with knee osteoarthritis and reported root mean square error (RMSE) values of 2.6°–2.7° for hip and knee joint angles.

Similarly, markerless motion capture systems provide an alternative to gait analysis that eliminates the need for wearable sensors or reflective markers [32]. Using a single camera and computer vision techniques enables spatio-temporal and kinematic parameters to be acquired in a more natural manner. Significant progress has been made in the field of Human Pose Estimation (HPE) in identifying joint points using Convolutional Neural Networks (CNNs) for accurate 2D pose detection [33,34], using a specific library such as OpenPose [35], MediaPipe [36,37], and PoseNet [38].

Several studies have explored the potential of markerless systems for estimating the kinematic parameters of the human gait cycle [39,40,41,42,43,44]. In particular, Molteni and Andreoni [39] aimed to assess the reliability of OpenPose in measuring the kinematics and spatio-temporal gait parameters in comparison to an optoelectronic system. Similarly, Hii et al. [40] conducted a comparative analysis of several pose estimation algorithms to identify the most suitable method for extracting spatio-temporal parameters. D’Antonio et al. [41,42] developed a system combining OpenPose with two cameras to measure lower-limb joint angles of one subject during gait. Similarly, Gupta et al. [43] employed MediaPipe Pose to estimate knee flexion and extension angles of one subject, comparing their results with those from Kinovea.

Although several studies have explored markerless pose estimation for gait analysis, to our knowledge, few studies have investigated the integration of MediaPipe with a single smartphone camera and its validation against wearable IMUs such as OPAL (APDM Wearable Technologies, Inc., Portland, OR, USA) within the specific experimental configuration adopted in this study, including the use of an 8 m walkway to capture complete gait cycles and a comparatively large cohort of healthy participants. The use of twenty-seven healthy subjects was primarily intended to validate the acquisition and estimation system under standardized conditions, minimizing potential confounding factors associated with pathological gait patterns. Moreover, the relatively large sample size, compared to those typically reported in the literature, allowed for a more robust and reliable assessment of the system’s performance.

Although we use existing tools (MediaPipe and OPAL Wearable Sensors), to our knowledge, these have been assembled and combined here for the first time, providing new insights into their integrated use.

In this context, we present a low-cost, user-friendly markerless method for assessing knee flexion/extension and ankle dorsiflexion/plantarflexion using MediaPipe with a single smartphone camera, uniquely validated against a dedicated IMU system on a comparatively large cohort of healthy participants, with the objective of providing a practical transferable framework for lower-limb kinematic analysis outside laboratory settings, emphasizing usability and real-world applicability.

## 2. Materials and Methods

### 2.1. Participants

Twenty-seven healthy volunteers (mean age: 26.61 ± 3.51 years; mean height: 168.47 ± 5.48 cm; mean weight: 69.33 ± 6.10 kg; mean BMI: 24.30 ± 4.10 kg/m^2^) took part in this study, including 15 women and 12 men. The study was conducted in a university laboratory, and participants were recruited on a voluntary basis. Their age, height, weight, and BMI were recorded. All participants provided informed consent to take part in the experiment in accordance with the Declaration of Helsinki.

### 2.2. Experimental Design

**Figure 1:**
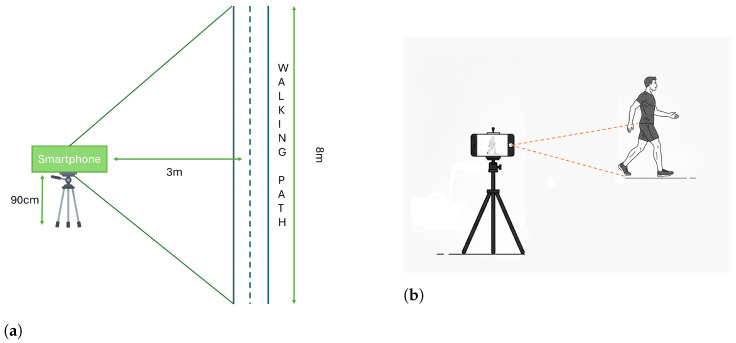
Experimental setup for video-based gait acquisition. (**a**) Top-view schematic of the acquisition layout: the smartphone is mounted on a tripod at a height of 90 cm and placed laterally at a distance of 3 m from the walking path, ensuring that the entire 8 m walkway falls within the camera’s field of view. (**b**) Side view of the recording configuration, showing the lateral placement of the camera to capture the subject’s full body silhouette in the sagittal plane, which is required for correct motion reconstruction.

The kinematic measurements were recorded using a standard smartphone with an average rate of 30 fps and a resolution of 1920 × 1080 pixels. The experimental setup involved a smartphone mounted on a tripod positioned at 0.9 m above the ground and laterally to the subject’s walking path. This height was selected in line with previous gait analysis and pose estimation studies, which typically report camera placements within a comparable range (approximately 0.7–1.3 m) [45,46,47]. Positioning the camera at 0.9 m allows approximate alignment with the participant’s pelvic region and center of mass, while maintaining full-body visibility within the field of view. The lateral placement ensured that the subject’s entire silhouette was captured, as complete body visibility is required for reliable motion reconstruction (see Figure 1). Subjects walked at a self-selected speed in a straight line parallel to the camera, which was placed laterally to the walkway to record movement in the sagittal plane. The camera was positioned to cover 8 m section of the walkway within its field of view. The recorded video data was transferred wirelessly from the smartphone to a computer. The acquisition process was synchronised at the beginning using a simple verbal cue, providing a common temporal reference. Since the wearable sensors provide processed kinematic parameters (e.g., maximum flexion, extension, and ROM) rather than continuous time-series data, this approach ensured practical alignment and allowed accurate comparisons between the HPE framework and the IMU-based measurements.

**Figure 2:**
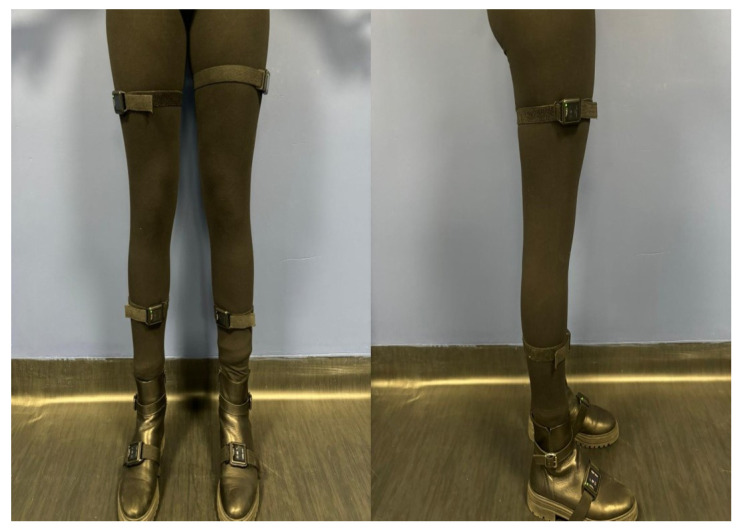
Position of the OPAL wearable sensors on the lower limbs to measure the knee and ankle joint angles.

To provide a benchmark for comparison with the markerless approach, subjects wore six OPAL sensors (APDM Wearable Technologies, Inc., Portland, OR, USA), attached to the foot, shank, and thigh of both lower limbs using straps to minimize relative movement between each sensor and the corresponding body segment (Figure 2). Sensors on the shanks were placed at mid-shank, approximately halfway between the ankle and knee joint centers, while sensors on the thighs were positioned over the muscle belly of the thigh, roughly corresponding to the midpoint of the segment.

Each IMU was equipped with a triaxial accelerometer, gyroscope, and magnetometer, and wirelessly synchronized with a laptop using the APDM Moveo Explorer software (APDM Wearable Technologies, Inc., Portland, OR, USA).

### 2.3. Camera Calibration

**Figure 3:**
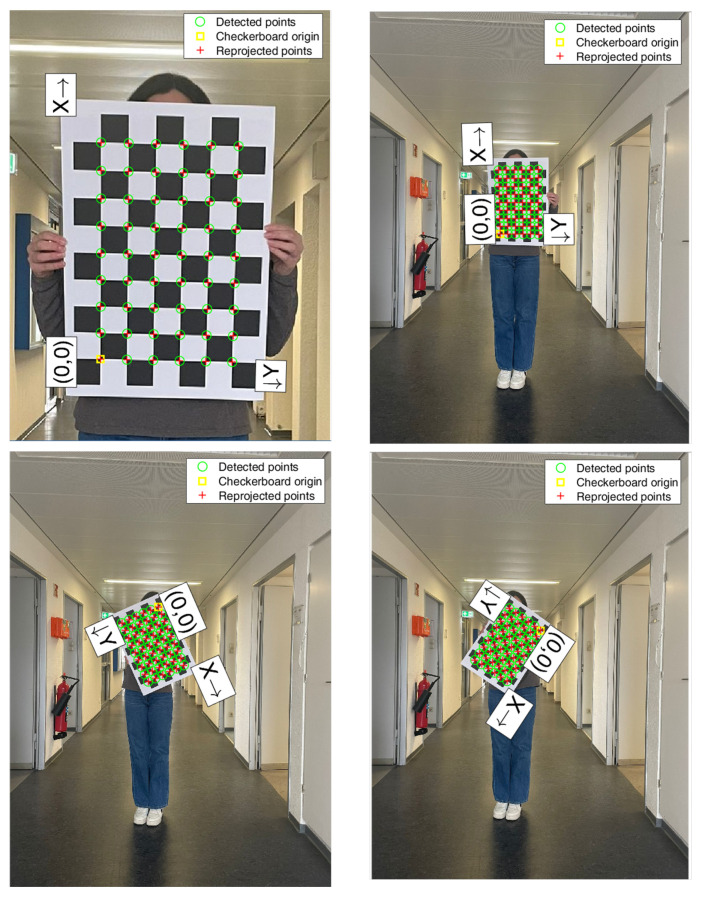
Example of camera calibration process using a checkerboard pattern. The four images show different orientations and positions of the checkerboard captured by a smartphone camera for intrinsic and extrinsic calibration. Green circles indicate the detected corner points, the yellow square marks the checkerboard origin (0, 0), and red crosses represent the reprojected points. The X and Y axes illustrate the local coordinate frame associated with the checkerboard.

All data were post-processed using MATLAB (vR2024b, The MathWorks Inc., Natick, MA, USA). The Single Camera Calibration app was employed for camera calibration to correct for any lens distortion present in the captured images. A series of 20 images, for three calibrations, of a planar 7 × 9 grid checkerboard pattern, consisting of black and white 55 × 55 mm squares, was captured by the camera while moving the pattern within the working volume (Figure 3). The checkerboard pattern images were imported into the Single Camera Calibration app (v.R2024b), which implements Zhang’s calibration algorithm [48]. The extrinsic and intrinsic parameters obtained through the calibration procedure were then applied to correct the images, allowing the 2D joint positions provided by MediaPipe.

### 2.4. Markerless Algorithms

**Figure 4:**
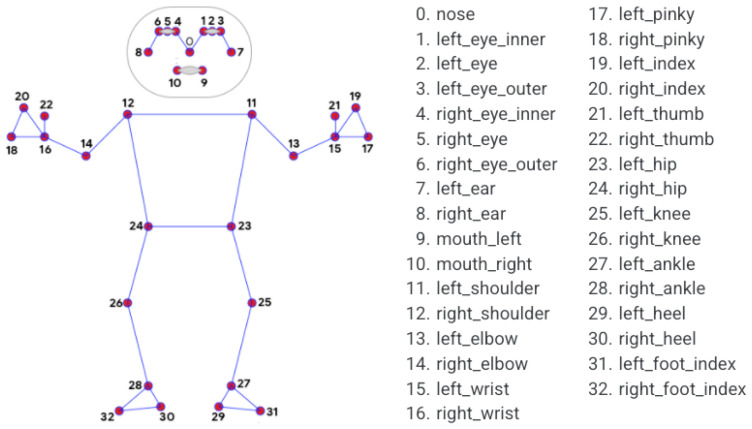
Standard landmark representation as defined by MediaPipe Pose: each red point corresponds to a key anatomical landmark, connected by lines reflecting the skeletal topology used for pose estimation. Image obtained from [37].

MediaPipe was used for HPE [36]. This open-source markerless video-based algorithm, developed by Google, was designed to estimate 2D coordination of the human joint in each frame of an image. MediaPipe detects in an image or video the body or region of interest, such as a person, face, or hands. Once detected, MediaPipe uses CNNs to estimate key points within that region and then tracks these key points across subsequent frames to analyze movements or poses. A specific component of the MediaPipe library, known as BlazePose (BZ), is tailored for highly accurate and efficient pose estimation [49]. BZ adopts a top–down strategy (detector/tracker) for the initial frame, and then switches to a bottom–up approach in subsequent frames, for subsequent frames, where key points are predicted solely by the tracker. The main strength of BZ is its lightweight design and fast execution, allowing it to run at a high frame rate on almost any device or hardware, including smartphones and CPUs. MediaPipe (v.0.10.14) with the BZ full model identified and extracted 33 distinct 2D landmarks across the human body, as illustrated in Figure 4.

### 2.5. Gait Parameter Extraction

**Figure 5:**
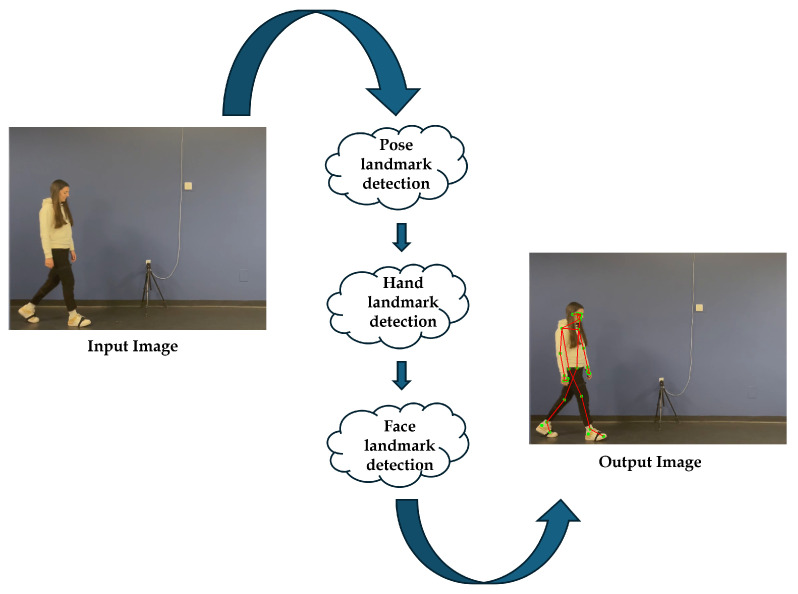
Workflow of the human motion capture by video using MediaPipe: starting with the original video, followed by the detection of keypoints and joint positions, and ending with the overlay of the MediaPipe skeleton on the video.

The proposed system employed a Python-based (v.0.10.14) solution running on a laptop to estimate lower limb kinematics through a markerless pose estimation model. The system processed walking videos recorded with a smartphone camera and calculated the movements of the knee and ankle (Figure 5).

It then outputted the kinematic parameters for each walking session in an Excel file for each healthy subject.

**Figure 6:**
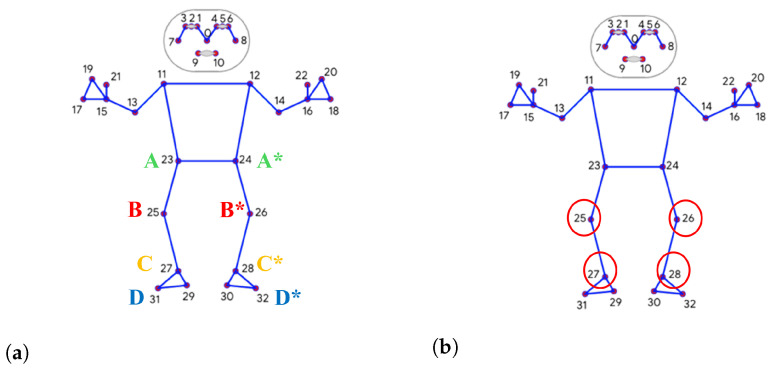
Representation of the human body using numbered landmarks for motion analysis. (**a**) MediaPipe keypoints considered for calculating joint angles of the right (A, B, C, D) and left (A*, B*, C*, D*) limbs; (**b**) the keypoints highlighted with red circles to indicate the angle joint of our interest.

To assess the flexion and extension of the lower limbs, specifically, the knee and ankle joints the sagittal plane the following landmarks are considered (Figure 6):

- Right Hip: defined as key point “23”
- Right Knee: defined as key point “25”;
- Right Ankle: defined as key point “27”;
- Left Hip: defined as key point “24”;
- Left Knee: defined as key point “26”;
- Left Ankle: defined as key point “28”;
- Right Toe: defined as key point “31”;
- Left Toe: defined as key point “32”.

Considering these key points, it was possible to define the knee flexion–extension and ankle dorsi-plantarflexion angles for the right and left limb (Figure 6). Three key points were considered for the calculation of each angle:

- Right Knee: defined as right hip (A) – right_knee (B) – right ankle (C)
- Left Knee: defined as left hip (A*) – left knee (B*) left ankle (C*);
- Right Ankle: defined as right knee (B) – right ankle (C) – right toe (D);
- Right Ankle: defined as right knee (B*) – right ankle (C*) – right toe (D*).

Using the coordinates of the hip, knee, ankle, and toe from the pose data, vectors were constructed to calculate the angles (Figure 6b).

For the knee angle, $\theta_{1}$ is given by the following equation:

(1) $$\theta_{1}=\arccos\left(\frac{\vec{AB}\cdot \vec{BC}}{|\vec{AB}\left|\cdot \right|\vec{BC}|}\right)\;,$$

where $\vec{AB}$ is the vector extending from the hip to the knee, and $\vec{BC}$ is the vector extending from the knee to the ankle.

For the ankle angle, $\theta_{2}$ is given by the following equation:

(2) $$\theta_{2}=\arccos\left(\frac{\vec{BC}\cdot \vec{CD}}{|\vec{BC}\left|\cdot \right|\vec{CD}|}\right)\;,$$

where $\vec{BC}$ is the vector extending from the knee to the ankle, and $\vec{CD}$ is the vector extending from the ankle to the toe.

### 2.6. Data Collection

To enhance data repeatability, each subject performed two walking sessions from right to left and two walking sessions from left to right. For each walking session, videos captured at least three gait cycles per subject. To ensure data robustness, the average across the three gait cycles of the knee flexion–extension angle and the average ankle dorsi-plantarflexion angle were calculated for each session. Additionally, since each subject walked twice for both the left and right limbs, the results of the two walks were averaged. For example, if “Subject 1” completed three gait cycles during the first walk from right to left, the average of the three maximum flexion values was calculated as *mean1*. The same calculation was repeated for the second walk, yielding *mean2*. Finally, the overall average, *meantot*, was computed as the mean of *mean1* and *mean2*. In detail, the main kinematic parameters were (i) the absolute maximum angles; (ii) the absolute minimum angles; (iii) the ROM, defined as the absolute difference between the maximum and minimum angles.

### 2.7. Statistical Analysis

Statistical analysis was conducted using IBM SPSS Statistics v.29 to evaluate and validate the reliability and robustness of the proposed system by comparing the kinematic gait outcomes obtained from wearable sensors with those from our system. Descriptive statistics were calculated to determine the mean and standard deviation of the kinematic parameters. To assess statistically significant main effects, paired-samples *t*-tests or Wilcoxon signed-rank tests were performed, depending on the normality of the data.

The correlation and absolute agreement between the two systems were evaluated using Spearman correlation coefficients (ρ) and inter-class correlation (ICC), respectively. The significance level for all analyses was set at 0.05. The performance of Spearman correlation and ICC was interpreted according to an established guideline that categorizes the results as poor (<0.500), moderate (0.500–0.750), good (0.750–0.900), and excellent (>0.900). Scatter plots of MediaPipe versus the OPAL wearable system were generated for the kinematic gait parameters for the means between the two sessions of each subject. Additionally, to evaluate and compare the accuracy and reliability of two measurement systems, Bland–Altman (BA) analysis was conduced in Matlab (v. R2024b, The Mathworks Inc., Natwick, MA, USA). In particular, the BA method was used to visually assess the agreement between the two measurement systems [50]. In the graphical representation of BA, the mean of the two measurements is plotted on the x-axis, while the difference between the measurements is plotted on the y-axis. To determine the limits of agreement, defined as the mean of the differences ±1.96 times the standard deviation (representing a 95% confidence interval), we first calculated the mean difference (bias) and then the standard deviation (SD) of the differences. This method allows for evaluating errors such as the distance of bias from zero, which indicates a consistent systematic error, and examining the width of the limits of agreement and any trends in data distribution around the bias. Potential issues include a fan-shaped spread within the limits of agreement (indicating heteroscedasticity), a non-random, systematic pattern in absolute values, or a linear trend in data points, which would suggest a systematic proportional error.

Finally, a Mean Absolute Error (MAE) was calculated as a performance metric:

(3) $$\;MAE=\frac{1}{N}\sum_{i=1}^{N}\left|x_{i}-\hat{x_{i}}\right|\;,$$

where $x_{i}$ and $\hat{x_{i}}$ represent the reference and estimated angle joint, respectively, and *N* is the number of observations.

## 3. Results

**Figure 7:**
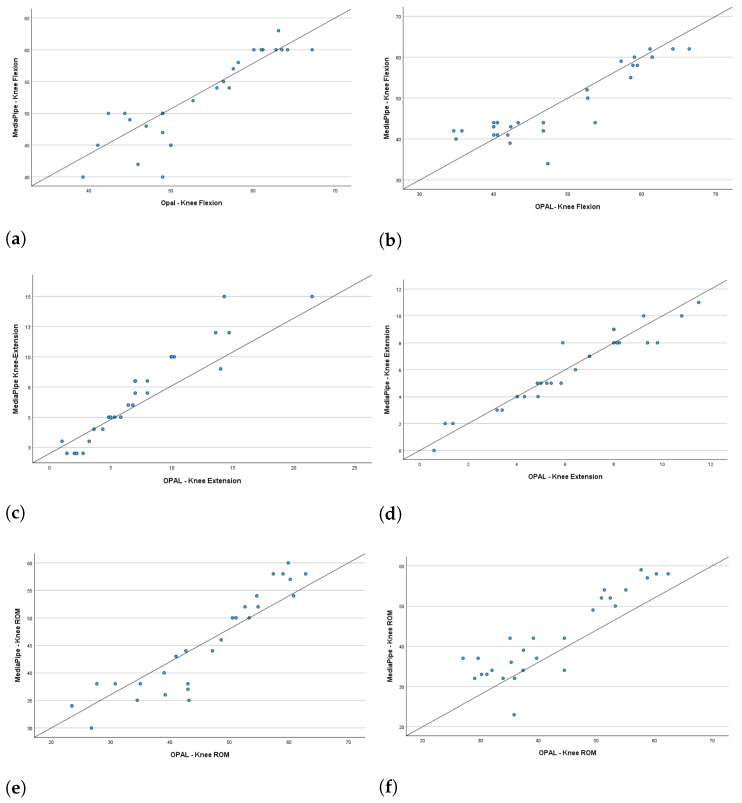
Scatter plots for the knee joint: (**a**,**b**) right and left knee flexion; (**c**,**d**) right and left knee extension; (**e**,**f**) right and left knee ROM.

**Figure 8:**
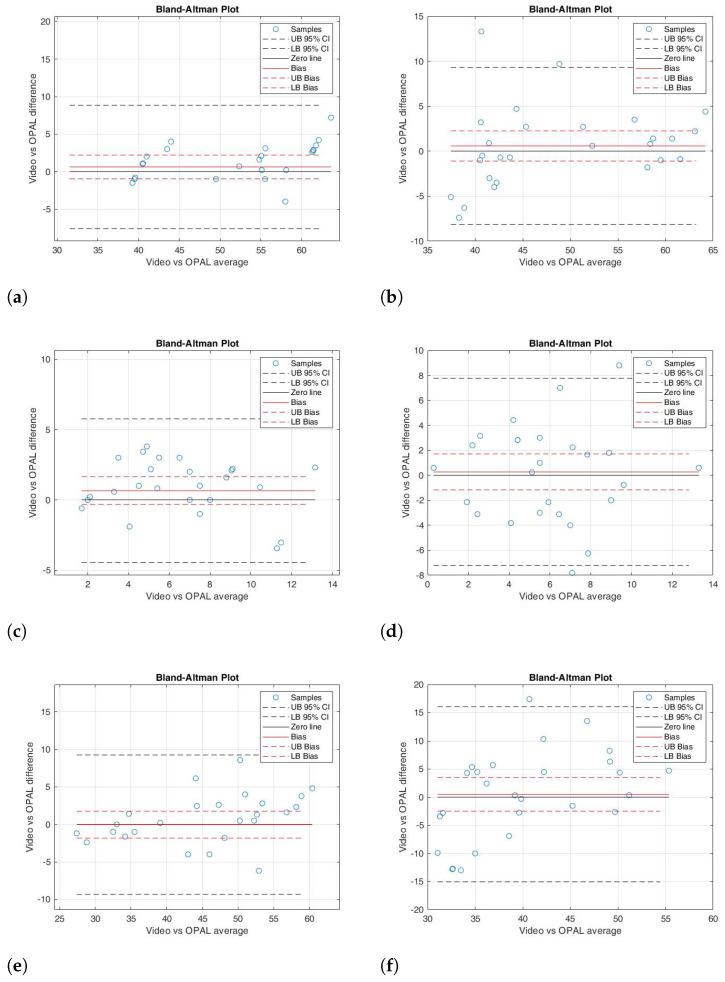
BA plot illustrating the agreement between the MediaPipe-video system and OPAL wearable sensors for knee flexion–extension and ROM for the right side (**a**,**c**,**e**) and the left side (**b**,**d**,**f**), respectively.

**Figure 9:**
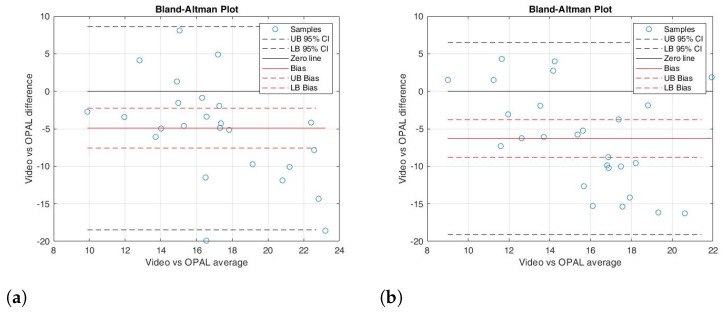

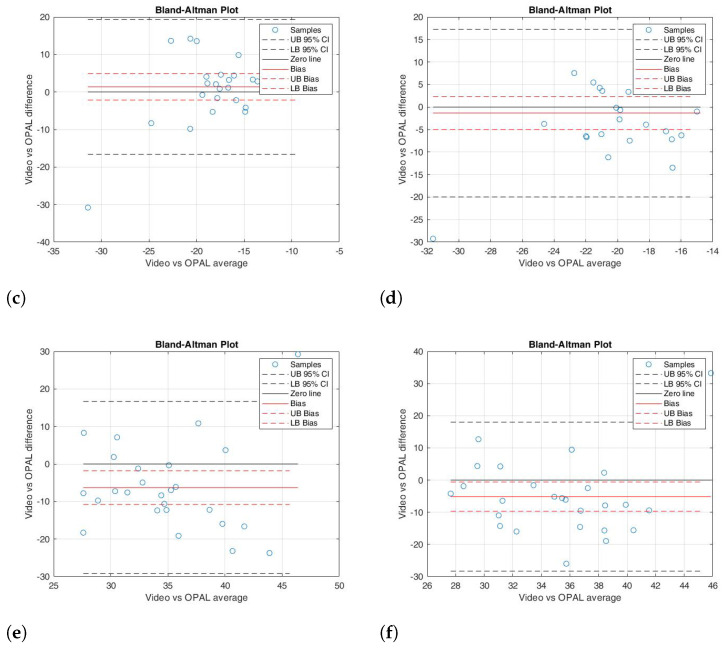
BA plot illustrating the agreement between the MediaPipe-video system and OPAL wearable sensors for ankle dorsi-plantarflexion and ROM for the right side (**a**,**c**,**e**) and the left side (**b**,**d**,**f**), respectively.

Given the non-normal distribution of the data, statistical significance was evaluated using the Wilcoxon signed-rank test. Table 1 presents the results, including the *p*-value, the ρ of the correlation coefficient, and the ICC. Additionally, it provides the mean and standard deviation for each kinematic parameter, both for the right and left sides, as well as the ROM. The BA analysis results for each kinematic parameter evaluated in the study are displayed in Table 2. These results are reported in terms of bias (the difference between the two measurement systems), the 95% lower and upper bounds of the bias, and the BA limits of agreement. The bias values were close to zero, and the bias limits, which contain the zero value, indicate a good agreement between the measured parameters. Figure 7, Figure 8 and Figure 9 depict the scatter plots for the knee joint, and the BA plots for both knee and ankle joints across all parameters. These parameters include right knee flexion, right knee extension, right knee ROM, left knee flexion, left knee extension, left knee ROM, right ankle dorsiflexion, right ankle plantarflexion, right ankle ROM, left ankle dorsiflexion, left ankle plantarflexion, and left ankle ROM.

## 4. Discussion

This study proposes a low-cost and user-friendly markerless motion capture method for gait analysis. The method consists of a single smartphone camera and a HPE framework, with OPAL wearable sensors by APDM Wearable Technologies serving as a benchmark for evaluating the kinematic parameters of twenty-seven volunteer subjects, providing additional validation of MediaPipe against a specific IMU system on a relatively large sample of healthy participants. It is designed to facilitate data collection in everyday settings using widely accessible tools, such as a standard smartphone. This approach is particularly suited for caregivers at home, who can record a walking session and share the video with clinicians, allowing remote assessment of a patient’s motor function or pathological gait patterns. This method has produced particularly promising results for the knee joint. This is of particular interest in applications aiming to study and identify biomechanical walking patterns useful for diagnosing knee injuries and conditions such as ligament damage, femoral syndrome and osteoarthritis [10]. Additionally, this approach may provide useful in investigating neurodegenerative conditions, where abnormalities in gait and alterations in joint kinematics serve as critical markers for early detection and tracking of disease advancement.

Among the assessed kinematic parameters, including knee flexion–extension and ankle dorsiflexion–plantarflexion, good agreement was achieved for knee flexion–extension.

In detail, the Wilcoxon test for knee flexion (right and left) showed a non-significant difference between the two instrumentations (*p*-value = 0.636 and 0.955, respectively); the correlation analysis was excellent for the right side (ρ = 0.916) and strong for the left side (ρ = 0.845). In addition, the ICC values were 0.828 for the right and 0.824 for the left (Table 1). The BA analysis revealed a small bias (0.61 and 0.58, respectively), with the 95% confidence interval containing the 0 value (Table 2). In the BA plot, the data points were randomly distributed around the 0 line (Figure 8a,b). These results indicated that there was strong agreement between the two measurement systems for knee flexion movement.

For knee extension (right and left), the paired data test showed slight significant differences (*p* = 0.042 and 0.049, respectively). However, the correlation analysis showed a good correlation (ρ = 0.862 and 0.845, respectively) and a moderate ICC (0.798 and 0.756, respectively) (Table 1). The BA analysis also showed a small bias of 0.66 for the right side and 0.27 for the left side (Table 2), with the zero line close to the limits of agreement (Figure 8c,d).

Similarly to the knee flexion results, the paired data test for ROM on both the right and left sides showed non-significant differences between the two systems (*p* = 0.274 and 0.586, respectively). Correlation analysis and the ICC indicated excellent agreement for the right side (ρ = 0.932, ICC = 0.839) and good agreement for the left side (ρ = 0.867, ICC = 0.920) (Table 1). The BA analysis showed a bias close to 0 (bias = 0.10 for the right side and 0.50 for the left side), with the 95% confidence intervals including 0 (Table 2). Additionally, the sample distribution followed a random pattern around the bias line (Figure 8e,f).

The scatter plots revealed a linear correlation between the kinematic gait parameters obtained from the MediaPipe system and those from the OPAL wearable system for the knee joint, based on the averages for each healthy subject (Figure 7).

Conversely, the agreement between the two instruments was not consistent across all gait kinematic movements. The paired data test showed a significant overall difference for the ankle joint (*p*-value < 0.05). Specifically, the Wilcoxon test revealed significant differences in dorsi-flexion for both the right and left sides (*p* = 0.001 for each). Furthermore, ROM also showed significant differences between the two instruments (*p* = 0.011 and 0.036, respectively). Nonetheless, no significant differences were found for plantarflexion on either side (right: *p* = 0.452, left: *p* = 0.475). As for the correlation between the two systems, the values were <0.5, indicating a low correlation (Table 1). Additionally, the weak agreement between the two instruments for ankle joint movements was further corroborated by ICC analyses, which showed values around 0.5. Regarding the BA analysis, there may be a constant systematic error, as indicated by the wide confidence intervals for both dorsiflexion and ROM on both sides. The BA analysis, shown in Table 2, confirmed the presence of a constant systematic error for the dorsiflexion and ROM, with significant biases (bias = −4.92, −6.30, −6.30, and −5.14, respectively) that were notably different from 0, and their 95% confidence intervals did not include 0, as depicted in Figure 9a,b,e,f. As for plantarflexion, the paired data test showed no significant difference for either the right or left side (*p*-value = 0.452 and 0.475, respectively), but a low correlation and ICC were reached (ρ < 0.5, and ICC= 0.564 and 0.524, respectively) (Table 1). In contrast, the BA plot showed a bias close to 0 for both sides (1.37 and −1.35, respectively) (Table 2), with the 0 line remaining within the agreement limits (Figure 9c,d).

Our results suggest that the proposed video-based, markerless method can capture knee joint kinematics, with a mean MAE of 4.10° ± 2.32° and 3.15° ± 3.10° for right and left knee flexion, respectively, and a MAE of 2.30° ± 2.01° and 3.12° ± 2.63° for right and left knee extension, respectively. The MAE for right and left knee ROM was found to be 4.55° ± 3.12° and 4.15° ± 3.01°, respectively. In accordance with Cerioli et al. [44], vision-based methods exhibited results similar to IMUs for knee joints, with MAE of $4.8^{\circ }\pm 4.3^{\circ }$. In contrast, ankle joint parameters showed lower agreement in most conditions, particularly for dorsiflexion and ROM, where both significant statistical differences and constant systematic errors were observed. Only ankle plantarflexion showed a closer agreement, indicating that certain ankle movements may be more reliably tracked than others using this approach. These results are in accordance with the study of Cerioli et al. [44], who higlighted the challenges of vision-based systems in measuring ankle joint motion. Similarly, our results, particularly those concerning the analysis of the knee joint in the sagittal plane in healthy adults, align with the findings of Molteni et al. [39], who employed OpenPose and a traditional optoelectronic system as the reference. Despite differences in methodology, both studies consistently indicate that markerless gait analysis can provide an accurate estimation of lower limb kinematics. Furthermore, our results are consistent in part with those obtained by D’Antonio et al. [41], who found that OpenPose underestimated the maximum knee flexion compared to IMU-based measurements, likely because of limitations in tracking the entire ROM during flexion. However, in our case, MediaPipe showed a reduced overestimation of knee parameters. In both studies, a similar trend emerged: the mean values of HPE system for right- and left-knee extension were lower than those obtained from the IMUs, supporting D’Antonio and colleagues’ observations [41]. Similarly, Gupta et al. [43] observed a correlation of 0.941 between the results obtained with Kinovea^®^ and MediaPipe, and an MAE of knee angles of 5.88°. Although Gupta et al. reported comparable results, our study uses wearable IMU sensors (OPAL APDM Wearable Technologies, Inc., Portland, OR, USA) as a benchmark. Unlike Kinovea^®^, which relies solely on 2D video analysis, IMUs provide direct 3D kinematic measurements with high temporal resolution. This allows for a more objective and quantitative validation of the markerless system. Finally, our study involved twenty-seven participants, providing a larger and more robust dataset than the limited sample in the Gupta et al. study [43].

Regarding the ankle joint, the mean MediaPipe values for dorsiflexion of right and left side were slightly greater than those measured by the OPAL system. Yamamoto et al. [51] reported comparable results when they compared OpenPose with 3D motion capture systems. Therefore, the results of our study highlight a significant difference in performance between knee and ankle joint measurements, probably because ankle movements are more complex or subtle. The reduced area of the ankle joint makes it more susceptible to inaccuracies and imprecise tracking in markerless pose estimation systems, and the position of the camera is a limiting factor. If we were to position the smartphone at ankle level, for example, we would not be able to capture the entire silhouette, resulting in failure to reconstruct it. In this context, in agreement with [39], a limitation of the current study may be related to the resolution and positioning of the smartphone camera. Indeed, previous studies have demonstrated that higher-resolution cameras can enhance accuracy and reliability [52]. Future work could explore using high-resolution cameras and optimising camera positioning to improve system performance.

To the best of our knowledge, the majority of scientific studies that have employed a markerless framework has been performed with a very limited number of participants or published data. In this context, our study, conducted on a group of twenty-seven healthy subjects, represents a comparatively larger and more robust dataset for preliminary validation, despite the homogeneity of the sample. It allowed us to perform a rigorous and controlled evaluation of the proposed markerless method, focusing specifically on its technological validity, accuracy, and reliability rather than on clinical outcomes, while reducing potential confounding factors associated with pathological gait variability. However, in line with the existing literature [39,53,54,55], it can be argued that markerless gait analysis systems can capture walking patterns not only in healthy individuals, but also in those with pathological gait patterns [39]. This supports the potential generalisability of such approaches. For this reason, future research will focus on testing the proposed method in more heterogeneous groups, including older adults and individuals with gait impairments. It will also be tested in real-world settings, such as domestic environments, to provide participants with a more comfortable and familiar setting and to better assess its applicability.

## 5. Conclusions

In conclusion, the study proposes a markerless method for gait analysis based on a single smartphone camera and a HPE framework, highlighting that their combination allows for portable, low-cost, real-time gait analysis with potential future implementation in embedded systems, increasing usability in both clinical and domestic settings. The approach shows promise for remote patient monitoring and for the study of musculoskeletal or neurodegenerative diseases. The results show that the markerless method provides excellent accuracy for the knee confirming the validity of the method for joint assessment, such as monitoring patients in post-surgical rehabilitation or with neurological, musculoskeletal, or orthopedic disorders. On the contrary, the assessment of ankle movements shows limited accuracy. The results confirm what has been observed in previous studies regarding the accuracy of markerless systems for the knee and the difficulties in detecting ankle movements.

## Figures and Tables

**Table 1:** Statistical comparison of kinematic parameters between MediaPipe and OPAL systems for both right and left sides.

Parameters (°)	MediaPipe	Opal	*p*-Value	ρ	ICC	MAE
**Right**						
Knee Flexion	50.87±7.76	49.18±14.47	0.636	0.916	0.828	4.10±2.32
Knee Extension	6.79±3.54	7.26±4.31	0.042	0.862	0.798	2.30±2.01
Knee ROM	44.08±9.57	41.87±14.47	0.274	0.932	0.839	4.55±3.12
Ankle DorsiFlexion	19.79±5.91	14.86±3.93	0.001	<0.500	0.478	7.80±4.15
Ankle PlantarFlexion	18.30±4.33	16.92±7.92	0.452	<0.500	0.564	5.50±2.01
Ankle ROM	38.08±7.70	31.78±7.80	0.011	<0.500	0.501	7.10±2.36
**Left**						
Knee Flexion	47.51±8.20	47.45±10.31	0.955	0.845	0.824	3.15±3.10
Knee Extension	6.40±3.22	6.80±3.40	0.049	0.845	0.756	3.12±2.63
Knee ROM	41.92±9.88	40.65±11.60	0.586	0.867	0.920	4.15±3.01
Ankle DorsiFlexion	18.78±5.55	12.48±3.19	0.001	<0.500	0.426	7.54±3.05
Ankle PlantarFlexion	18.81±4.20	20.17±7.32	0.475	<0.500	0.524	6.41±3.10
Ankle ROM	37.79±7.11	32.64±7.70	0.036	<0.500	0.487	6.94±4.23

Notes: ICC = Inter-Class Correlation; ρ = Spearman correlation coefficient; ROM = Range of Motion; MAE = Mean Absolute Error.

**Table 2:** Limits of Agreement for Kinematic Parameters (Bland–Altman Analysis) for both right and left sides.

Parameters (°)	Bias	LB_B	UB_B	LB_LA	UB_LA
**Right**					
Knee Flexion	0.61	−0.97	2.20	−7.62	8.85
Knee Extension	0.66	−0.32	1.64	−4.50	5.76
Knee ROM	0.10	−1.83	1.74	−9.25	9.33
Ankle Dorsiflexion	−4.92	−7.58	−2.26	−18.46	8.62
Ankle Plantarflexion	1.37	−2.15	4.90	−16.61	19.37
Ankle ROM	−6.30	−10.80	−1.80	−29.23	16.64
**Left**					
Knee Flexion	0.58	−1.11	2.26	−8.15	9.31
Knee Extension	0.27	−1.17	1.72	−7.20	7.85
Knee ROM	0.50	−2.49	3.49	−15.05	16.06
Ankle Dorsiflexion	−6.30	−8.81	−3.78	−19.10	6.50
Ankle Plantarflexion	−1.35	−5.00	2.30	−19.96	17.26
Ankle ROM	−5.14	−9.69	−0.60	−28.32	18.02

LB_B and UB_B = Lower and Upper Bound of Bias, respectively; LB_LA and UB_LA = Lower and Upper Limits of Agreement, respectively.

## Data Availability

The data presented in this study are available on reasonable request from the corresponding author. The data are not publicly available due to privacy and ethical restrictions.

## Abbreviations

The following abbreviations are used in this manuscript:

BA	Bland–Altman
BMI	Body Mass Index
BZ	BlazePose
CNNs	Convolutional Neural Networks
HPE	Human Pose Estimation
ICC	Interclass Correlation Coefficient
IMUs	Inertial Measurement Units
MAE	Mean Absolute Error
ROM	Range of Motion
RMSE	Root Mean Square Error
